# Including animals in sociology

**DOI:** 10.1177/00113921211065492

**Published:** 2021-12-29

**Authors:** Yasmin Koop-Monteiro

**Affiliations:** The University of British Columbia, Canada

**Keywords:** Animals, environment, environmental sociology, human–animal relations, social-ecological networks, Animaux, environnement, relations homme-animal, réseaux socio-écologiques, sociologie de l’environnement

## Abstract

How do we include animals in sociology? Although sociology’s initial avoidance of the nonhuman world may have been necessary to the field’s development, recent scholarship – within mainstream sociology, environmental sociology and animal-centred research – is helping expand the field’s horizons. With a focus on variety, this article reviews four key paths that researchers are taking to include animals in their research: (1) studying interspecies relations, (2) theorizing animals as an oppressed group, (3) investigating the social and ecological impacts of animal agriculture and (4) analysing social-ecological networks. This review shows how applying – and innovating – existing social theories and research methods allows researchers to include animals in their analyses and will be relevant to a variety of scholars, including mainstream and environmental sociologists, animal-focused researchers and social network analysts, to name a few.

## Résumé

Comment inclure les animaux dans la sociologie ? Bien que l’évitement initial du monde non humain par la sociologie ait pu être nécessaire au développement de celle-ci, des études récentes – au sein de la sociologie traditionnelle, de la sociologie de l’environnement et de la recherche centrée sur les animaux – contribuent à élargir les horizons du champ sociologique. En mettant l’accent sur la variété, cet article passe en revue les quatre voies principales que les chercheurs suivent pour inclure les animaux dans leurs recherches: (1) l’étude des relations entre les espèces, (2) la théorisation des animaux en tant que groupe opprimé, (3) l’étude des répercussions sociales et écologiques de l’élevage, et (4) l’analyse des réseaux socio-écologiques. Nous montrons ainsi en quoi le fait d’appliquer – et d’innover – des théories sociales et méthodes de recherche existantes permet aux chercheurs d’inclure les animaux dans leurs analyses et sera utile à toute une série de chercheurs, y compris les sociologues traditionnels et les sociologues de l’environnement, les chercheurs axés sur les animaux et les analystes de réseaux sociaux, parmi tant d’autres.

## Resumen

¿Cómo incluimos a los animales dentro de la sociología? Aunque la evitación inicial de la sociología del mundo no humano puede haber sido necesaria para el desarrollo de la disciplina, los estudios recientes, dentro de la sociología convencional, la sociología ambiental y la investigación centrada en los animales, está contribuyendo a expandir los horizontes de la disciplina. Poniendo el enfoque sobre la variedad, este artículo revisa cuatro vías fundamentales que los investigadores están siguiendo para incluir a los animales en sus investigaciones: (1) el estudio de las relaciones entre especies, (2) la teorización de los animales como un grupo oprimido, (3) el estudio de las consecuencias sociales y ecológicas de la agricultura animal, y (4) el análisis de las redes socio-ecológicas. Esta revisión muestra cómo el hecho de aplicar, y de innovar, las teorías sociales y los métodos de investigación existentes permite a los investigadores incluir a los animales en sus análisis, y es relevante para una variedad de investigadores, incluidos los sociólogos generales y ambientales, los investigadores centrados en los animales y los analistas de redes sociales, entre otros.

## Introduction

Nonhuman animals, and our relationships with them, have played a critical role in the development of society. In the pre-agricultural age, hunted animals provided a means for survival,^1^ and human–nonhuman relationships (particularly with canines) offered humans companionship and protection (Horard-Herbin et al., 2014). As humans began domesticating other animals, horses provided transportation, while oxen and others pulled ploughs and moved heavy cargo for farming and construction (Kalof, 2007). Even today – in the post-industrial age where machines have surpassed the limits and labour power of nonhuman animals – human society is shaped by its relationships with both domesticated and wild animals.^2^ Our interactions with these animals within the food system, alone, bear significant influence on climate change (Poore and Nemecek, 2018) and the development of zoonotic diseases (Espinosa et al., 2020). However, due to the history of the field of sociology, nonhuman animals have largely been absent within sociological theory and research.^3^ Some have attributed sociology’s animal exclusion to George Herbert Mead, whose focus was on culture and symbolic meaning (Alger and Alger, 2003; but see Wilkie and McKinnon’s (2013) animal-inclusive reading of Mead). Others point to Talcott Parsons, who ‘seem[ed] to take it for granted that sociality is the privilege of humans’ (Halewood, 2014: 113) and that theory of social action cannot explain nonhuman action.

Thanks, in part, to Jane Goodall (1971) and the many animal researchers who followed her (e.g. Bekoff, 2002; Faust, 2011; Irvine, 2007), studies of nonhuman animals helped establish that many have complex social structures – and that culture and social action are not uniquely human traits. Nevertheless, because sociology was trying to carve a place for itself while steering clear of racist and eugenic explanations of reality, it focused on the social and cultural aspects of human society (Carter and Charles, 2018). Indeed, Buttel (2002) considers classical theorists’ exemption of human society from the influence of nature ‘to have been more a necessary step in the development of a nondeterministic sociological tradition’ (p. 43). Thus, by the early 20th century, ‘The door was firmly shut . . . on beings and objects defined as unable to participate in meaning making and cultural production’, and animals became ‘defined as the concern of biology rather than sociology, despite their importance to the constitution of societies’ (Carter and Charles, 2018: 84–85). At first necessary, such bracketing of human society from the nonhuman world would slowly begin to change by the later half of the 20th century (Bryant, 1979), although conceptualizations of nonhuman animals, and their inclusion in sociological research, remain marginal within the field (Peggs, 2012).

In this article, we explore key efforts to address sociology’s nonhuman exclusion. We begin with a brief review of environmental sociology and its attempts to challenge the mainstream human-focused paradigm. From there, we traverse four major and intersecting paths^4^ that researchers within and outside sociology are taking to include nonhumans in their social analyses. The first broadly explores human–nonhuman interactions. The second approaches animals as an oppressed group. The third considers the social and ecological impacts of the animal agriculture industry. Finally, the fourth, and perhaps most innovative research current, uses a social network analysis perspective to study structural relationships between human and nonhuman entities. In reviewing a diversity of intersecting research paths, this article aims to provide scholars with a roadmap on how to begin incorporating nonhuman animals into their analyses.^5^ Furthermore, by spotlighting recent innovations in concepts (e.g. nonhuman agency), theory (e.g. oppressed groups and alienation theories) and methods (e.g. social-ecological networks), it seeks to inspire researchers to develop additional theories and methods to further integrate animals into sociological research.

## Bringing nature into sociology

Soon after the growth of the modern environmental movement in the 1960s (Rootes, 2014), the field of sociology welcomed a new and distinctive subfield: environmental sociology. Inspired, in part, by Rachel Carson’s (1962) *Silent Spring* and the Apollo missions’ first images of Earth, a new ecological consciousness took hold. Quite rapidly, beliefs in the inseparability of society from nature, and the need for profound structural change, became the spirit of the time. This was also true for sociologists looking to broaden the field’s gaze and create an *environmental* sociology that takes society’s relationship with the environment as its bedrock. Chief to its foundation was the New Ecological Paradigm (NEP) presented by Catton and Dunlap (1978), which challenged the Human Exemptionalist Paradigm (HEP) that the authors observed in mainstream sociology.

Central to HEP is the prominence of human culture that, given its dynamism, is believed to help humans overcome natural limits of the environment (Catton and Dunlap, 1978). NEP, on the contrary, highlights humanity’s reciprocal relationship with nature and the biological limits of the environment (Catton and Dunlap, 1978). By implication, sociological research would need to include the nonhuman world in its analyses.

While NEP challenged the dominant sociological paradigm, this was not an attack on sociological theories. It was, instead, a call for contemporary sociologists to broaden their focus and include society’s relationship with the nonhuman world. Indeed, resurrections of the discipline’s founding theorists reveal their careful attention to society’s relationship with the environment. Marx spoke of the modern human’s alienation from nature, as well as industrial society’s disrupted social-ecological ‘metabolism’ (Foster, 1999). Weber emphasized nature’s influence on the organization of society, such as the ancient Bedouin peoples’ nomadic lifestyle given the harsh environmental conditions of North African and Middle Eastern deserts (Foster and Holleman, 2012). Durkheim borrowed heavily from Darwin’s evolutionary theory (Catton, 2002) and ascribed causative roles to population density and resource scarcity (Buttel, 2002). And Simmel recognized society’s reciprocal relationship with the environment, presenting nature as an actor with its own ‘rights’ to exert influence over human-made structures (Gross, 2000). Revolutionary at the time, environmental sociology, with its focus on society–environment relationships, is now a well-established area within sociology.

More recently, however, some have challenged environmental sociology’s NEP as insufficient in its inclusion of nature. Tovey (2003), for example, criticizes environmental sociology for failing to meaningfully incorporate nonhuman animals. Without recognizing animals’ unique individuality, she argues, sociologists limit themselves to perceiving animals only as ‘risks’ or ‘natural resources’ (Tovey, 2003). They also, inadvertently, uphold the human exemptionalism that NEP so strongly criticized. In response to both mainstream and environmental sociology’s insufficient attention to human–animal relationships, important efforts to include nonhuman animals within the field have emerged. Here, we explore four key ways to include animals in sociology.

### Analysing interspecies relations

The first research path focuses on *human–animal relationships and their implications for human society*. For most of human history, humans have freely interacted with other animals in various contexts, including relations of friendship, companionship, and hunting for survival. From the beginning of the Neolithic era, however, human–nonhuman relationships became more controlled and instrumental with the shift from hunter-gatherer societies to the domestication of animals. Whereas subsistence hunters would seek to maintain proper and respectful relations with animals and nature,

[S]tarting about 10,000 years ago, the human relationship with wild animals was transformed. Domestication changed this relationship into one of dominance and control as humans took on the role of master. Animals – no longer wild – became classified as property, and were considered items to be owned and exchanged. Today, domesticated animals can be bred, controlled, abused, or killed as the owner desires. (DeMello, 2012: 67–68)

Furthermore, as more animals were being used for animal agriculture, they began to disappear from public view. With growing industrialization in the 18th and 19th centuries, nonhuman animals were increasingly kept in warehouses and stockyards where, in Fordist assembly-line fashion, they were gathered, killed and turned into meat. While, in some cases, spectators could observe this process (Pacyga, 2015), further industrialization of animal agriculture in the 20th century rendered these animals invisible, as they were increasingly crowded in factory farms with no public access.

Although most humans in contemporary society will never meet individual members of these domesticated species, they are nevertheless part of society. In fact, it is generally agreed that these nonhuman animals’ domestication was only possible because of their ‘sociality and ability to communicate’ with humans (Tovey, 2003: 212). In her book *When Species Meet*, Haraway (2008) writes, ‘Earth’s beings are prehensile, opportunistic, ready to yoke unlikely partners into something new, something symbiogenetic. Co-constitutive companion species and coevolution are the rule, not the exception’ (p. 220). In a detailed analysis of the close bonds that can form between human and nonhuman animals, Haraway uses the biological analogy of ‘reciprocal induction’ to describe how members of different species learn to communicate and develop trust, influencing each other in the process (see also Irvine and Cilia, 2017). Although her analysis focuses on a particular species (dogs), the implications regarding interspecies communication can extend to animals used in the animal agriculture industry. Within the industry, ‘gaining the trust’ of these animals, and training them to ‘respond to nudges from a stick’, is emphasized to make their handling and slaughter easier (Irvine and Ellis, 2010: 26). Critically, exploitation does not negate possible feelings of kinship. Here, Ellis (2020: 9, 13) evidences how ranchers experience contradictory caretaking and exploiting roles, as they engage in both ‘fathering’ *and* ‘domination’, both ‘mothering’ *and* eventual ‘trafficking’ of those they breed (see also Wilkie’s (2010) in-depth exploration of the paradoxical human-and-farmed-animal relationship).

New forms of human–nonhuman interactions have also developed with respect to wild animals. One example is ‘ecotourism’, where humans seek out encounters with nature and wild animals. In *Facing the Wild*, Bulbeck (2005) discusses the growth of ecotourism – including bird-watching and swimming with dolphins. Here, she suggests ‘embodied experiences with actual animals’ are key to the experience and more impactful than just reading about them (Bulbeck, 2005: xix). However, a growing body of research on animals in media suggests that strategically planned animal portraits and wildlife images can evoke significant empathy and interest among humans (Whitley et al., 2020). Studies of accidental encounters with wild animals also reveal the subtle ways that these nonhumans shape human experiences in both natural and human-built settings. For example, unplanned encounters with wolves, birds, and bears can enhance humans’ hiking and skiing experiences (Arbieu et al., 2020; Stoddart, 2011). Simialrly, Ávila and Ernstson (2019) show how encounters with scorpions emerging from bathtub drains can force city-dwellers into fearful confrontations – and conscious co-inhabitation – with wild nonhuman animals.

Finally, a unique form of animal advocacy, called ‘bearing witness’, has emerged, which incorporates human–nonhuman interactions. Here, activists standing outside slaughterhouses lock eyes with slaughter-bound animals – and document these animals’ conditions – as slaughterhouse trucks drive past (Purdy and Krajnc, 2018). From its beginnings outside Canadian slaughterhouses,^6^ bearing witness is now practised worldwide. Thus, studies of how animal advocates engage various movement tactics around the world (e.g. Cherry, 2016) offer researchers another opportunity to consider nonhuman animals’ place in society. Social psychological research on concern for animals (Dietz et al., 2017) offers another avenue for exploring the status of animals and animal advocacy across cultures.

Although these and other researchers have made significant progress in including animals in their studies, an important aspect of human–nonhuman interactions – animal agency – has been difficult to capture. To help address this issue, Carter and Charles (2018) update the sociological concept of agency to be more inclusive of nonhumans. Specifically, they distinguish one’s *capacity to act* (which is an attribute) from *agency* – which is informed by context. ‘On this view of agency’, they argue, ‘non-human animals are agential beings’ who are incorporated into social relations ‘on the basis of difference and inequality’ (Carter and Charles, 2018: 93). Their agency is, therefore, involuntarily modified by the various situations they find themselves in with humans – ‘sheep do not decide when they are to be slaughtered; chickens do not decide to live in cramped and insanitary battery cages; wolves and pheasants are unaware of the shooting season’ (Carter and Charles, 2018: 92; see also Chan and Harris, 2011; Healey and Pepper, 2021; Latour, 1996 for various perspectives on *who* – or even *what*, in Latour’s case – is capable of exercising agency). To properly account for nonhuman agency, Blattner et al. (2020) suggest researchers study contexts (such as sanctuaries) in which animals maintain sufficient agency in their relations with humans. Otherwise, a lack of *realized* agency (due to violence, deprivation and confinement) could be mistaken for a lack of *potential* agency, obscuring efforts to exercise agency that fall outside clear acts of resistance (such as attacks, escape and insubordination; e.g., Hribal, 2011). The remaining three sections proceed from, and intersect with, this broader theme of interspecies relations.

### Studying animals as an oppressed group

Another way in which sociologists are incorporating nonhuman animals in their research is by studying them as *an oppressed group*. This research current is analytically diverse and grounded in normative concerns. In his essay ‘Experiments on Animals’, psychologist Richard Ryder (1971) introduces the term ‘speciesism’ to define prejudice against other species and in favour of one’s own species, comparable, in this sense, to racism, sexism or ableism. Taking a sociological perspective, Nibert (2003) clarifies that speciesism is not merely prejudice – it is an ideology that, similar to ‘racism, sexism, classism, and other ‘isms’, is based on ‘a set of socially shared beliefs that legitimates an existing or desired social order’ (p. 8). Here, Nibert (2003) recrafts minority group theory – which he calls ‘oppressed groups’ theory – to highlight the ‘entangled oppression of humans and other animals’ (p. 5).

According to Nibert (2003), because most social scientists agree that oppression is socially arranged (not natural or necessary), and many accept that different oppressions are related or entangled (e.g. Patricia Hill Collins’ (1998) work on the intersections of race, class and gender), social theory is primed to consider both human and nonhuman oppression. ‘An oppressed group’, Nibert (2003), notes, ‘shares physical, cultural or economic characteristics and is subjected, for the economic, political and social gain of a privileged group, to a social system that institutionalizes its exploitation, marginalization, powerlessness, deprivation or vulnerability to violence’ (p. 8). By avoiding human-specific and depoliticized concepts (as the term ‘minority’ may, by default, define dominant groups as ‘normal’ or ‘typical’), such a definition of oppressed groups brings the potential for deeper understanding of how oppression works in society today, and throughout history (Nibert 2003).

In *Animal Oppression and Human Violence*, Nibert (2013) traces some of the historical roots of human and nonhuman oppression. Reviewing human history from the Neolithic Revolution to today, he finds important links between the domestication of animals with human inequality and violence. Similar to Engels^7^ (1884: 29–30), Nibert attributes the rise in gender inequality to the development of organized hunting and animal domestication, which lead to unequal divisions of labour, the devaluation of women’s labour and male control of surplus wealth. This surplus wealth (widely measured by one’s ownership of animals) necessitated the creation of a ‘warrior class’ and ‘priestly class’ to defend and manage this wealth (Nibert 2013). Similarly, the domestication of horses and cows in the ‘Old World’ allowed humans to travel long distances, facilitating lengthy wars as cows often marched along soldiers ‘as “meat on the hoof”’ (Nibert, 2013: 36). Pre-colonial Americas, on the contrary, did not have horses and cows (and pigs and chickens, etc.) that colonizers brought, and so its wars may have been shorter in duration (Nibert, 2013: 71–74). Fast forward to today, and many humans are still suffering due to modern animal exploitation as factory farms pollute nearby communities (Son et al., 2021), developing countries devote vast areas of land to grow food for animals consumed mostly in developed countries (Sun et al., 2020) and zoonotic diseases continue to compromise public health (Espinosa et al., 2020).

Similarly, Tovey (2003) argues that animals are ‘invisible’ within sociology. Tackling environmental sociology in particular, Tovey (2003: 197) notes how nonhumans are primarily thought of in terms of ‘biodiversity’ or ‘species’, with the consequence that sociology largely ‘absorbs animals into “wild nature,” with virtually nothing to say about the huge numbers’ of domestic, service or ‘functional animals’ (bred for food, scientific experiments, transportation and entertainment). Noting the public’s widespread interest in animals, Tovey calls on environmental sociologists to better incorporate nonhumans into their analyses. For example, she highlights Raymond Murphy’s (1994) concept of ‘environmental classes’ as applicable to many animals, as his definition does not require self-conscious ‘awareness of class position . . . [so that even] foetuses are included’ (Tovey, 2003: 206). In addition to critiquing mainstream sociology’s exemptionalist paradigm, Tovey problematizes environmental sociology’s alternative ecological paradigm, which characterizes humans as ‘exceptional’ and has heretofore obscured nonhumans’ experiences of oppression (Tovey, 2003: 209). She therefore argues that researchers must

[I]ntroduce into sociology the recognition that we are not alone in the world, that other animal species also exist, have similar environmental experiences to our own, and are in many cases included within significant social relationships. (Tovey, 2003: 210)

In the spirit of Tovey’s proposed paradigm, researchers have developed new avenues to incorporate animals into analyses of society. Here, Adams (2018), notes how human/nonhuman females suffer under systems of male dominance. Reproductive autonomy, for example, is at stake for both humans *and* nonhumans, as nonhuman females’ reproductive systems are disproportionately exploited for milk, eggs and birthing new animals into existence. Similarly, Kheel (2008) highlights how sport hunters’ discourses often frame violence against women and the nonhuman world as a male right of passage. Studies of domestic violence also evidence the link between violence against pets and women’s risk of abuse (e.g. Barrett et al., 2020), while neighbourhoods with higher rates of slaughterhouse employment (compared to other employment types) appear to experience higher rates of violent crimes like sexual assault and rape (Fitzgerald et al., 2009). Deckha (2012) also spotlights how race and culture can intersect with animal oppression, as certain groups of humans have historically categorized others as ‘less than human’ for instrumental purposes, and the consumption of animals (both as food and as entertainment) has been equated with wealthier classes. In addition, Kim (2015) and Ko (2019) emphasize that due to the intertwined nature of racial and animal oppression, true progress on either issue requires advocates to attend to both injustices. Similarly, recent work taking a decolonizing perspective highlights the links between Indigenous and nonhuman oppression (including centuries of land dispossession for animal agriculture) and how incorporating Indigenous legal orders recognizing animal personhood may be key to reconciliation (Deckha, 2020; Montford and Taylor, 2020). Finally, Taylor (2017) underlines how disability and animal oppression are entangled – particularly in how ableism serves to justify oppressive treatment of those deemed physically or cognitively ‘inferior’.

### Studying the animal agriculture industry

Another key area of analysis surrounds society’s treatment of animals bred for food and its *impacts on humans, animals and the environment*. This path takes a ‘sector’ focus, often guided by materialist concerns within environmental sociology (York and Longo, 2017). Particular attention is given to how animal agriculture has changed since the 1950s via ‘interrelated processes of industrialization and mechanization, the consolidation of industries, the concentration of animals into feedlots, and the intensification of both the cropland used for animal feed and the production of farm animals themselves’ – which Gunderson and Stuart (2014) refer to as ‘industrial animal agribusiness’ (p. 55). Given the concentration of nonhuman animals within the industry, Gunderson and Stuart (2014) argue that ‘studying the consequences of industrial animal agribusiness provides an ideal entry point for environmental sociologists to begin incorporating animals into their analyses’ (p. 54; see also Kalof and Whitley’s (2021) materialist analysis of other domestic, wild and urban-dwelling animals).

Early Frankfurt School theorists – including Horkheimer, Adorno and Marcuse – were among the first modern social theorists to note the plight of farmed animals (Gunderson, 2014). Inspired, in particular, by Marx’s^8^ critique of capitalist exploitation of humans, animals and nature, these Frankfurt theorists problematized the domination and exploitation of the nonhuman world as sowing the seeds for the domination and exploitation of humans (Foster and Clark, 2016). For them, the ‘chain of domination starts with humanity’s material exploitation and conceptual degradation of animals’ so that the same ‘coldness’ and instrumentality that justify society’s treatment of animals (for scientific experiments, entertainment and slaughter) can quickly transfer to its treatment of fellow humans (Gunderson, 2014: 290).

More recently, social scientists have extended Marx’s concept of alienation to include nonhuman animals. As Benton (1993) argues, ‘the pathological distortions . . . which Marx attempts to capture in his concept of “estrangement,” or “alienation,” are in important respects paralleled in the modes of life imposed upon [farmed] animals’ (p. 42). As Dickens (1996) notes,

[A]nimals have now been ‘specialized’ . . . some cattle are for beef and some for milk. And some chickens are for humans to eat while others are raised for the production of eggs. The ‘species-being’ and ‘natural-being’ of animals are being treated as disaggregated wholes. (p. 63)

According to Dickens (1996), the ‘results can be horrendous’ as animals are removed from their natural environments, confined and mutilated to support the conditions of modern animal agriculture (p. 63). Similar to the de-humanizing effects of exploitative labour on humans, anthropologist Barbara Noske (1997: 18–21) argues that animals exploited for food have been ‘de-animalized’ as they are separated from their offspring, their capacity for free productive activity and nature. Indeed, recent analyses extending alienation to nonhuman animals have generated important insights on the welfare of cows, pigs and chickens (Gunderson, 2013) and the limitations of technological advancements in addressing animal suffering within the industry (Stuart et al., 2013).

Others have extended Marx’s later work on the emergent rifts between society and nature, given animal agricultural production (Foster, 1999). Foster and Magdoff (1998), for example, draw attention to soil fertility issues and pollution generated by industrial animal agriculture, which creates more solid waste than the surrounding soil can safely absorb. With respect to marine environment, Clausen and Clark (2005) find several important rifts generated by industrial fishing practices, including increased pollution and marine life depletion. Echoing climate research on animal agriculture’s impact on global carbon and nitrogen cycles (e.g. Poore and Nemecek, 2018), Gunderson (2011) shows that modern animal agriculture’s greenhouse gas (GHG) emissions, and its vast deforestation for pasture and animal feed, are driving climate change, eliminating carbon sinks and destroying habitats (thus threatening species extinctions). Similarly, other Marxian critiques of capitalist production, like Treadmill of Production (Schnaiberg, 1980), draw attention to the industry’s vast resource withdrawals (land, water) and inputs (air, water and soil pollution) (Gunderson and Stuart, 2014). Returning to the early Frankfurt School theorists, Stuart and Gunderson (2020) highlight Horkheimer, Adorno and Marcuse’s insights on how a ‘capitalist ethos’ and ‘domination ideology’ serve to justify and normalize nonhuman exploitation.

Contrary to Marxian analyses of the environment, modernization theories focus on social and technological innovations that may help bring humans back in balance with the nonhuman world. Ecological Modernization (Mol and Spaargaren, 2000; Spaargaren and Mol, 1992), for example, posits that economic growth without environmental destruction is possible. While applications of ecological modernization both within and outside of animal agriculture provide some evidence of increased efficiency, economic returns are nevertheless tied to negative industry impacts, including significant land use and GHG emissions for meat production (Horlings and Marsden, 2011; Jorgenson and Clark, 2012). Indeed, researchers note that because of the global industrialization and consolidation of feed production, animal farming and slaughter, the animal agriculture industry’s ecological impacts are often ‘complex’ and ‘hard to quantify’ (Jay and Morad, 2006: 477). Another modernization theory, Reflexive Modernization (Beck, 1992), focuses on how growing risks can trigger substantial change in how society and industry are organized. Research taking this perspective evidences the industry’s ‘anti-reflexivity’ or resistance to change (Bos and Grin, 2008; Stuart and Worosz, 2012), as well as news media’s lack of reflexivity in communicating industry-related risks (Capurro, 2020).

### Analysing social-ecological networks

**Figure 1.**
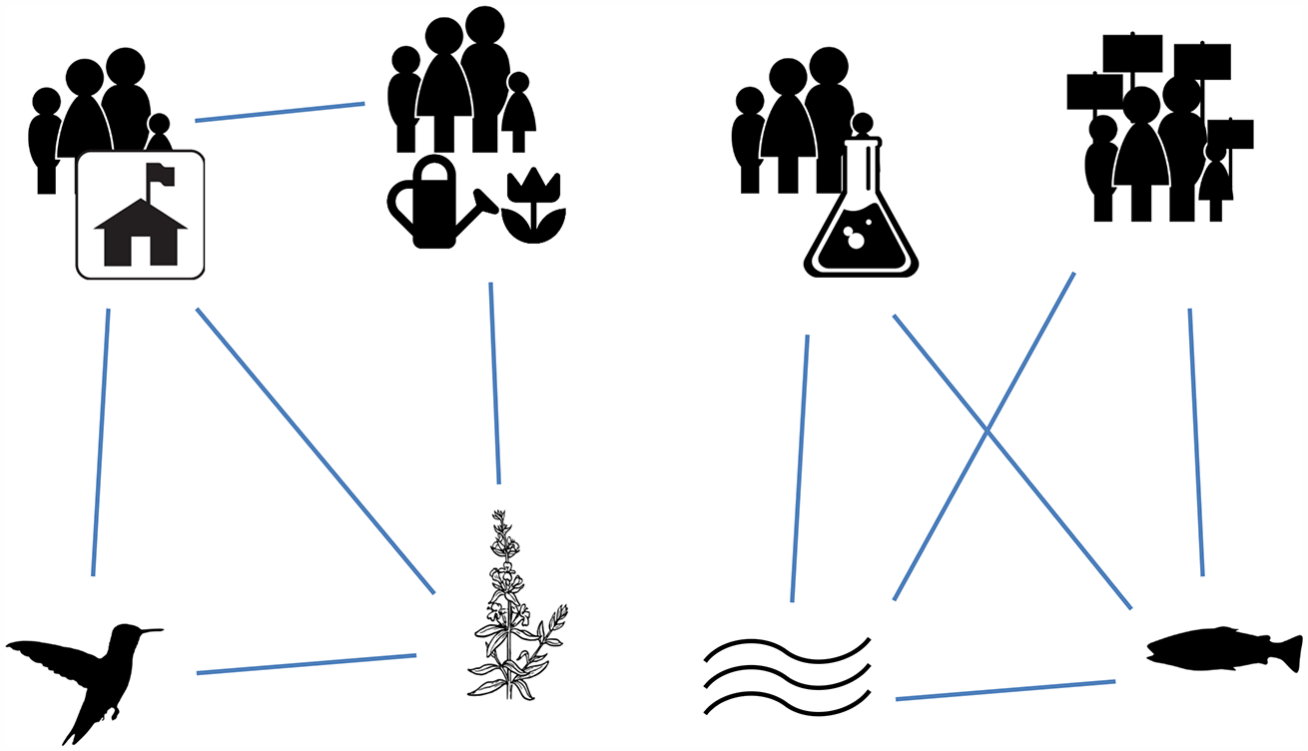
Visual depiction of two social-ecological networks showing ties within and across social and ecological domains. On the left, we see a network of human entities (park managers and community gardening groups) and nonhuman entities (pollinators and plant species). Here, nonhuman entities are connected (via mutualistic relation) and human entities are connected (e.g. via communication, thus facilitating collaboration). On the right, we see a network of human entities (scientists and environmental groups) and nonhuman entities (water bodies and aquatic life). Here, nonhuman entities are connected (via wildlife–habitat relation), while human entities are unconnected (despite common interests, thus impeding collaboration).

For sociologists taking a social networks perspective, the new area of *social-ecological networks* – with its structural focus on human–nonhuman interactions – offers a unique way to include nonhuman animals (see Figure 1). Briefly, social network analysis is a paradigm offering theoretical and methodological tools to ‘study social relations and their structuring’ (Prell, 2012: 1). Specifically, it studies *social structures as social networks* – as ‘a set of actors (nodes) and a set of relationships [ties] connecting pairs of these actors’ (Tindall and Wellman, 2001: 266; see also Borgatti, Everett, and Johnson, 2018; Scott, 2017, for complementary introductions). Whereas traditional social network analysis focuses on relationships between human actors (e.g. communication or collaboration between individuals and organizations), social-ecological networks also incorporate ecological entities (e.g. animal species, ecosystem components) and the relations between them (e.g. ecological relationships like predation, social-ecological relationships like ecosystem management) (Bodin et al., 2017, 2019). Importantly, within social-ecological networks, humans’ ties are often significant *because of* their ties to animals, habitats, and other nonhuman entities (e.g. border-crossing migratory pathways that necessitate collaboration between ecosystem managers).

In their introductory discussion of social-ecological networks, Cumming et al. (2010) propose three ways to study relationships between social and ecological entities, depending on the research question. The first is to analyse social and ecological networks separately (e.g. ecosystem manager network and ecosystem components network) to infer potential implications (e.g. disconnected managers in densely connected ecosystems). The second is to assign attributes of one network to another – by assigning social attributes to ecological nodes (e.g. social significance of certain habitat patches; Bodin et al., 2006) or ecological attributes to social nodes (e.g. ecosystem knowledge held by certain individuals; Bodin and Norberg, 2005). The third is to incorporate both social and ecological nodes into a single network (mapping all social–social, ecological–ecological and social–ecological relationships at once; e.g. Barnes et al., 2019). As an alternative to this third approach, one might include both types of nodes but only analyse relationships attached to one node type (e.g. examining social actors’ social–social and social–ecological ties, without articulating the ecological–ecological ties between nonhuman nodes (see Kluger et al., 2020; Sayles et al., 2019, for up-to-date reviews of studies using varying levels of social-ecological network articulation).

While research on social-ecological networks often aggregates individual animals into species (e.g. fish species; Barnes et al., 2019) or groups of species (e.g. seabirds; Bodin et al., 2017), this does not necessarily lead to the nonhuman invisibility that Tovey (2003) observes in mainstream and environmental sociology (see discussion on animals as an oppressed group for details). In fact, humans are frequently aggregated into groups (e.g. organizations; Ernstson, 2011), institutions (e.g. laws; Cumming et al., 2020), human actions (e.g. fishing strategies; Yletyinen et al., 2018) and human stressors (e.g. climate change; Zhao et al., 2018). In addition, nonhuman aggregation is often done for practical reasons, as collecting data on specific nonhuman ties may be impractical or unnecessary for the research question at hand. In the case of subsistence fishers, for example, their ties to fish are labelled according to the fish species they target (e.g. Barnes et al., 2020). Furthermore, in terms of affective human–nonhuman ties, narrative research on humans’ interactions with marine mammals, for example, indicates that humans can develop a generalized affection towards all members of a species (DeMares, 2000; Yerbury and Weiler, 2020).

Social-ecological networks can also be analysed qualitatively. Traditional forms of qualitative social network analysis incorporate a variety of approaches, including interviews, observational and archival data analysis, and can employ the many theories used by mainstream sociologists (Domínguez and Hollstein, 2014; Hollstein, 2014). This is also true of social-ecological network analysis. Here, interviews (Berman et al., 2004), expert knowledge (Zhao et al., 2018) and textual analyses of archival data (Ekstrom and Crona, 2017) may be used to map social and/or ecological network ties.

For example, in their analysis of urban ecosystems and ecosystem managers, Ernstson et al. (2010) combine ecological data of pollinator–plant species interactions with qualitative interviews and text analysis to map the various relations between environmental groups and their nonhuman foci. Qualitative analysis can take a more central role in social-ecological network research as well. Centring how knowledge about the environment is constructed, Ernstson (2013) applies actor–network theory and value articulation theory to ethnographic, interview and textual analyses on the importance of city parks – and their nonhuman inhabitants – to communities. More broadly, Ernstson (2013) demonstrates the value of qualitative social-ecological network analyses when studying how humans relate to parks, animals and other aspects of the nonhuman world.

## Conclusion

Since its ecological reckoning in the 1960s, the field of sociology has made important progress in its capacity to produce insights on human society’s relationship with the nonhuman world. Recent efforts to bring animals into sociology have produced new and exciting developments that take this project further. With a focus on variety, this article reviewed four key ways to incorporate nonhuman animals into sociological research: (1) exploring interspecies relations, (2) studying animals as an oppressed group, (3) investigating the impacts of the animal agriculture industry and (4) analysing human–nonhuman interactions as social-ecological networks. Of course, this should not be taken as an exhaustive review of the literature. Instead, this article is meant to illustrate some of the key ways in which social scientists are bringing nonhumans into their research. By spotlighting existing theoretical (e.g. oppressed groups theory, alienation theory), conceptual (e.g. nonhuman agency) and methodological (e.g. social-ecological network) innovations, this article aims to inspire researchers to innovate and extend additional theories, concepts and methods to further integrate animals into sociological research. Ultimately, tackling the discipline’s nonhuman exclusion – and including animals in sociological research – reflects a growing understanding of human society’s interdependence with the nonhuman world. It also reflects a commitment to a more comprehensive sociology that acknowledges human society’s significant relationships with its fellow nonhuman co-inhabitants.

## Notes

1. Importantly, many foraging societies relied on plants for the bulk of their nutrition (Park et al., 2016), while hunting would remain central to some settled societies (Norgaard, 2019).

2. The terms ‘wild’ and ‘domesticated’ are used for ease of understanding. However, more critical terms like ‘free-living’ and ‘domesecrated’ are applicable (Nibert, 2013).

3. Exceptions include Bryant’s (1979) call to include animals in sociology, Chase’s (1980, 1991) research on nonhuman behaviour to understand dominance hierarchies and mobility processes and Latour’s (2010) actor–network theory.

4. I use ‘paths’, ‘currents’ and ‘ways’ loosely because the four categorizations of research explored here are not mutually exclusive. Multiple ‘paths’ (e.g. ‘animals as oppressed’ and ‘interspecies relations’) can be applied within the same analysis, and there is much overlap between them. As such, the four categorizations can be more usefully thought of as ‘ideal types’ – deliberate simplifications and exaggerations for ease of understanding – than as rigidly defined procedures.

5. This review does not represent the complexity of the literature. It is limited to four themes to help frame some of today’s major efforts to include animals in social research and is by no means exhaustive.

6. Animal Save. Bearing Witness. Available at https://thesavemovement.org/bearing-witness/ (accessed 11 February 2021).

7. Engels’ (1884) *Origin of the Family, Private Property and the State* was based upon Marx and Engels’ observations of anthropologist Lewis Henry Morgan’s (1877) research in *Ancient Society*.

8. Marx criticized industrialized animal agriculture, noting how ‘In these prisons animals are born and remain there until they are killed off’ (International Institute of Social History Marx-Engels Archive Sign. B. 106, 209, 336, as cited in Foster, 2016: 15; Saito, 2016: 62).
